# Crystal structure and Hirshfeld surface analysis of an erbium(III) coordination polymer based on a 2,3,5,6-tetra­bromo­benzene-1,4-di­carboxyl­ate ligand

**DOI:** 10.1107/S2056989026010698

**Published:** 2026-10-20

**Authors:** Suwadee Jiajaroen, Kittipong Chainok

**Affiliations:** a Division of Science and Mathematic Faculty of Science and Technology Rajamangala University of Technology Tawan-ok Bangpra Sriracha Chonburi 20110 Thailand; b Thammasat University Research Unit in Multifunctional Crystalline Materials and Applications (TU-MCMA) Faculty of Science and Technology Thammasat University Khlong Luang Pathum Thani 12121 Thailand; Katholieke Universiteit Leuven, Belgium

**Keywords:** crystal structure, coordination polymer, erbium(III), carboxyl­ate

## Abstract

The title polymeric compound, poly[[tri­aqua­(μ_3_-2,3,5,6-tetra­bromo­benzene-1,4-di­carboxyl­ato)hemi(μ_2_-2,3,5,6-tetra­bromo­benzene-1,4-di­carboxyl­ato)erbium(III)] trihydrate], {[Er(C_8_Br_4_O_4_)_1.5_(H_2_O)_3_]·3H_2_O}_*n*_ or {[Er(Br_4_bdc)_1.5_(H_2_O)_3_]·3H_2_O}_*n*_ (**1**), was hydro­thermally synthesized and structurally characterized. The asymmetric unit contains one Er^III^ cation, one and a half Br_4_bdc^2–^ dianions, three coordinated H_2_O mol­ecules, and three uncoordinated solvent H_2_O mol­ecules. The central Er^III^ cation is seven-coordinated within a distorted capped trigonal-prismatic geometry. The coordination sphere is defined by four carboxyl­ate oxygen atoms from four Br_4_bdc^2–^ ligands and three aqua oxygen atoms. The Br_4_bdc^2–^ ligand occupies a general position, and acts as a *μ*_3_-bridge linking three symmetry-related Er^III^ cations. It coordinates *via μ*_3_-κ*O*:κ*O′*:κ*O′′* mode, using its carboxyl­ate oxygen atoms to extend into a two-dimensional polymeric sheet parallel to the *bc* plane. Meanwhile, the remaining Br_4_bdc^2−^ ligand is situated on a crystallographic inversion centre and adopts a *μ*_2_-κ*O*:κ*O′* coordination mode linking the sheets into a three-dimensional network. The cohesion of the three-dimensional supra­molecular framework is enhanced by classical O–H⋯O hydrogen bonds. The crystal packing is further consolidated by inter­molecular Br⋯Br and Br⋯O halogen bonding inter­actions. The analysis of the Hirshfeld surface reveals that the primary contributions arise from Br⋯Br (23.5%) and O⋯H/H⋯O (20.8%) contacts. Additional contributions include Br⋯H/H⋯Br (13.2%), H⋯C/C⋯H (12.5%), Br⋯C/C⋯Br (8.4%), Br⋯O/O⋯Br (7.0%), and O⋯O (5.3%) inter­actions.>

## Abstract 2

The crystal structure and Hirshfeld surface analysis of a new three-dimensional erbium(III) coordination polymer based on 2,3,5,6-tetra­bromo­benzene-1,4-di­carboxyl­ate ligand are reported.

## 1. Chemical context

Lanthanide coordination polymers (LnCPs) have been widely studied and their potential applications, including electronic, optical, and magnetic properties arising from the unique 4*f* electronic configurations of *Ln*^III^ ions (Bünzli, 2010 ▸; Capelo *et al.*, 2025 ▸; Shiels *et al.*, 2026 ▸). Unlike transition-metal ions, *Ln*^III^ ions exhibit high and variable coordination numbers together with a wide range of coordination geometries. They possess robust *Ln*—O bonds and a hard Lewis acid character, resulting in a strong affinity for hard donor atoms, particularly oxygen-containing ligands (Cui *et al.*, 2012 ▸; Saraci *et al.*, 2020 ▸; Costanzo *et al.*, 2026 ▸). These characteristics make *Ln*^III^ ions attractive building units and provide flexibility in the assembly of structural diversity (Bünzli, 2014 ▸; Boone *et al.*, 2024 ▸). Besides, the selection of ligands plays a crucial role in constructing diverse topologies and properties. Aromatic polycarb­oxy­lic acids are widely employed as organic linkers in the design and construction of CPs. In particular, terephthalic acid (H_2_bdc) and its functionalized derivatives display versatile coordination modes spanning monodentate, chelating, bridging, and chelating–bridging fashions, yielding structurally diverse networks upon self-assembly (Li *et al.*, 2014 ▸; Jiajaroen *et al.*, 2022 ▸; Nisa *et al.*, 2024 ▸). Among these derivatives, 2,3,5,6-tetra­bromo­benzene-1,4-di­carb­oxy­lic acid (H_2_Br_4_bdc) is structurally significant; the four bulky, electronegative bromine substituents drastically alter the steric and electronic environment of the central aromatic core, significantly influencing both the primary coordination spheres and the secondary supra­molecular inter­actions. The presence of bromine substituents may influence the opportunities for inter­molecular halogen-related inter­actions, particularly Br⋯Br and other halogen-containing contacts. These may play a crucial role in crystal packing and structural stabilization. Herein, we report the synthesis and crystal structure of a new erbium(III) CP, namely, poly[[tri­aqua­(μ_3_-2,3,5,6-tetra­bromo­benzene-4-di­carboxyl­ato)hemi(μ_2_-2,3,5,6-tetra­bromo­benzene-1,4-di­carboxyl­ato)erbium(III)] trihydrate] (**1**). The assembly of the polymeric framework and the supra­molecular inter­actions of **1** are thoroughly elucidated and examined. Furthermore, Hirshfeld surface analysis provides a qu­anti­tative evaluation of inter­molecular contacts and crystal packing inter­actions of **1**.
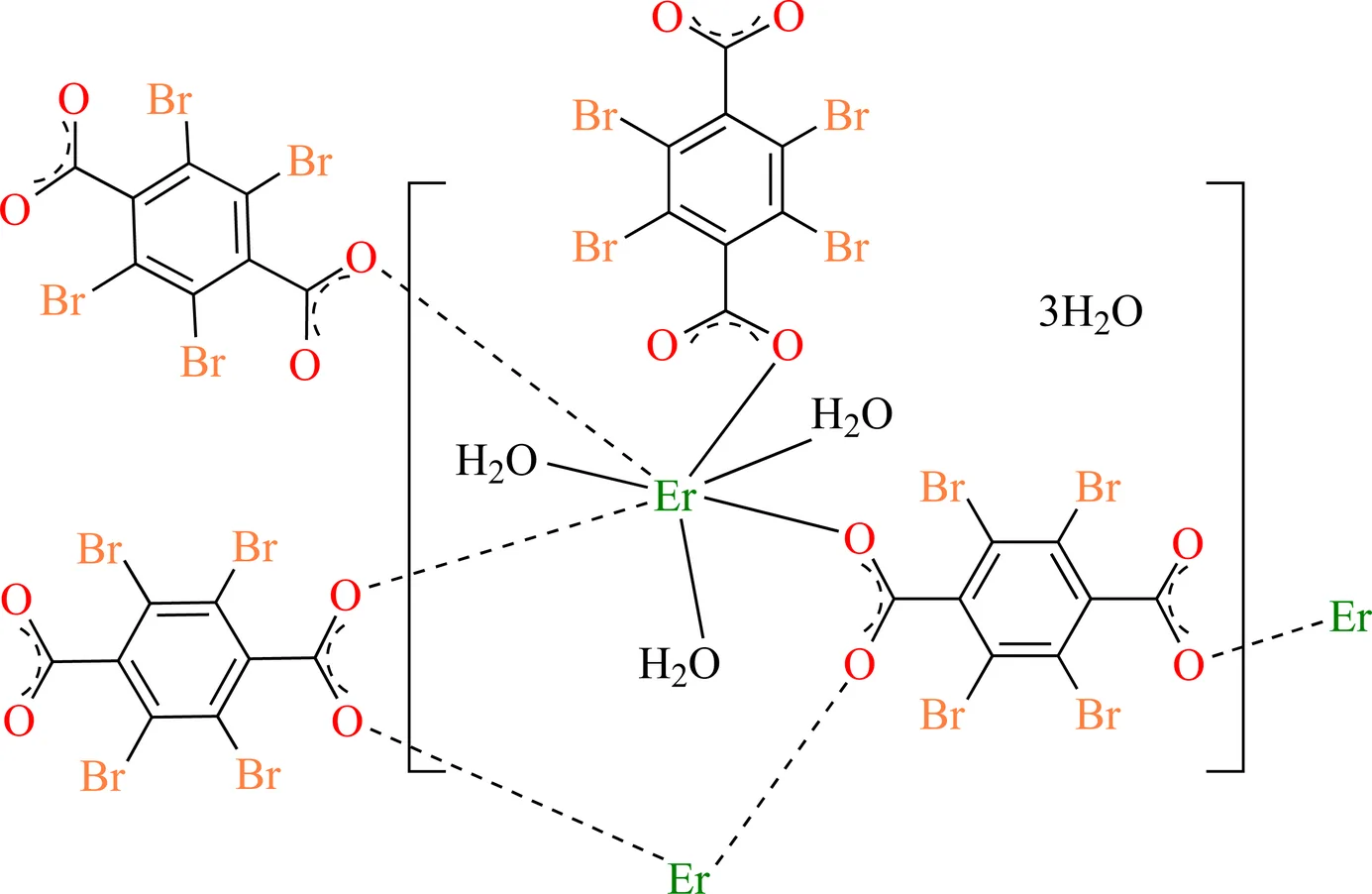


## 2. Structural commentary

**Figure 1:**
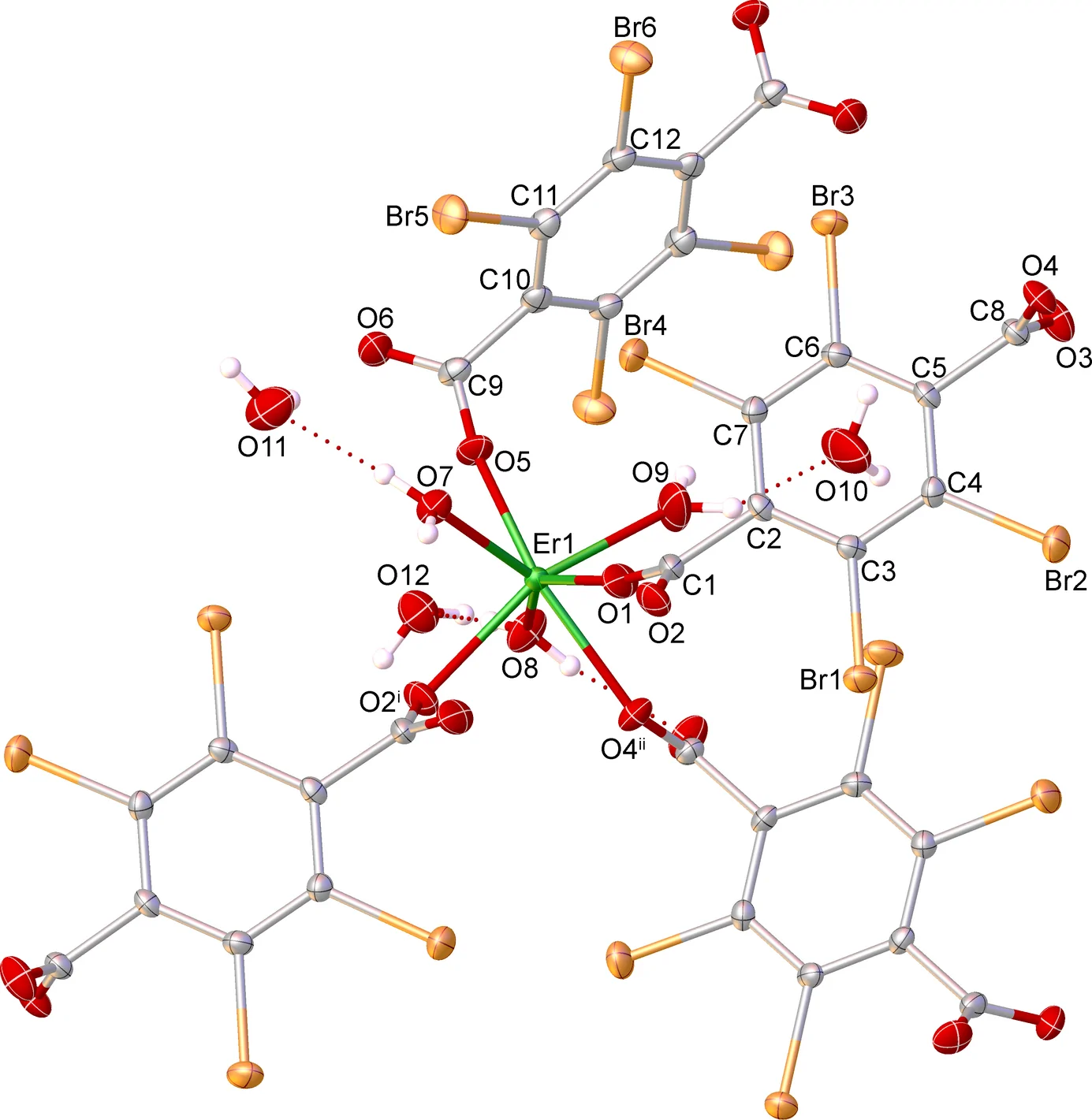
Mol­ecular structure of **1**, showing the asymmetric unit with the atom-labeling scheme and the coordination environment of the Er^III^ ion. Displacement ellipsoids are drawn at the 40% probability level. Symmetry codes: (i) 1 − *x*, 1 − *y*, 2 − *z* and *x*, ½ − *y*, ½ + *z*.

**Figure 2:**
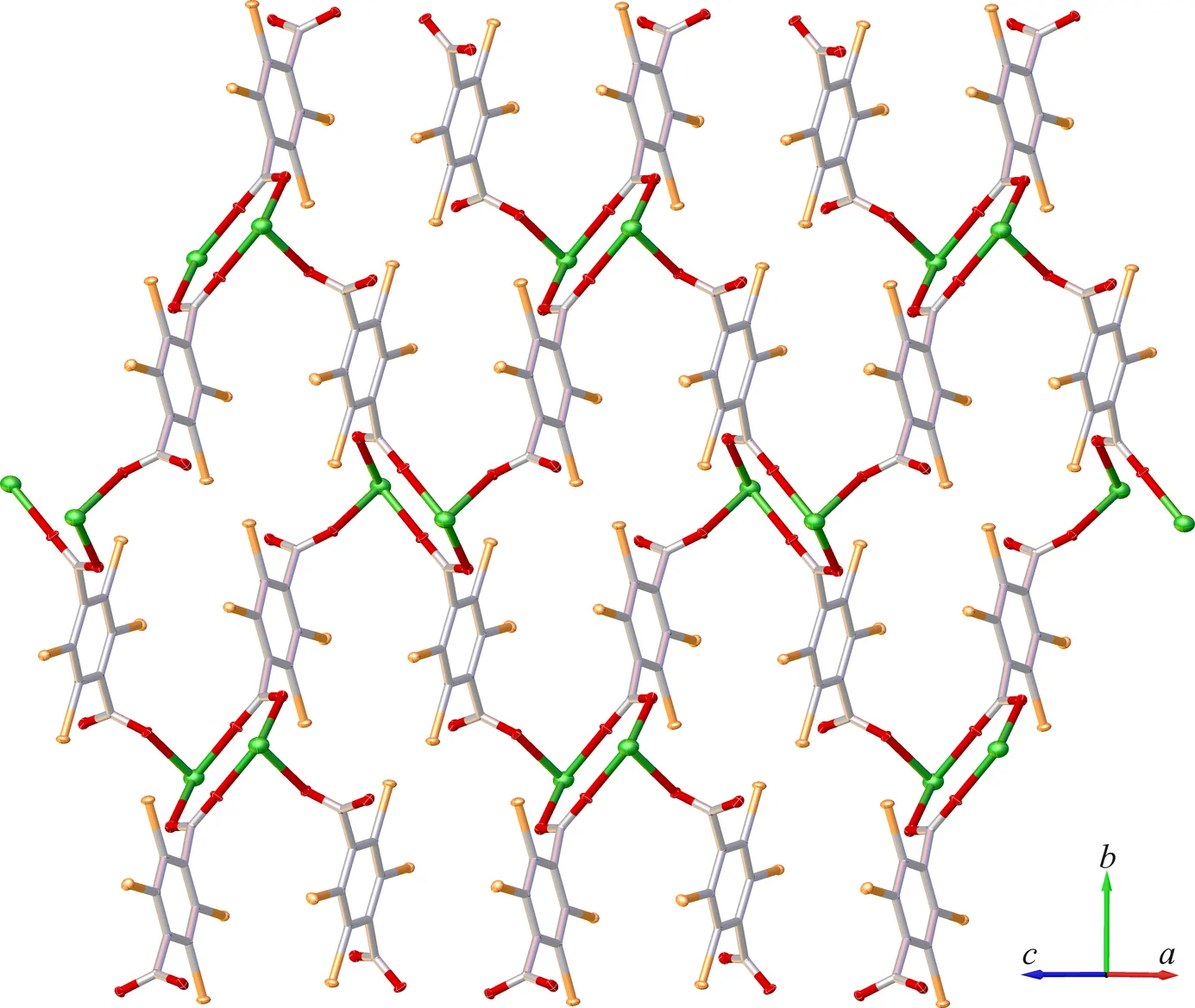
View of the two-dimensional sheet structure of **1** in the *bc* plane.

**Figure 3:**
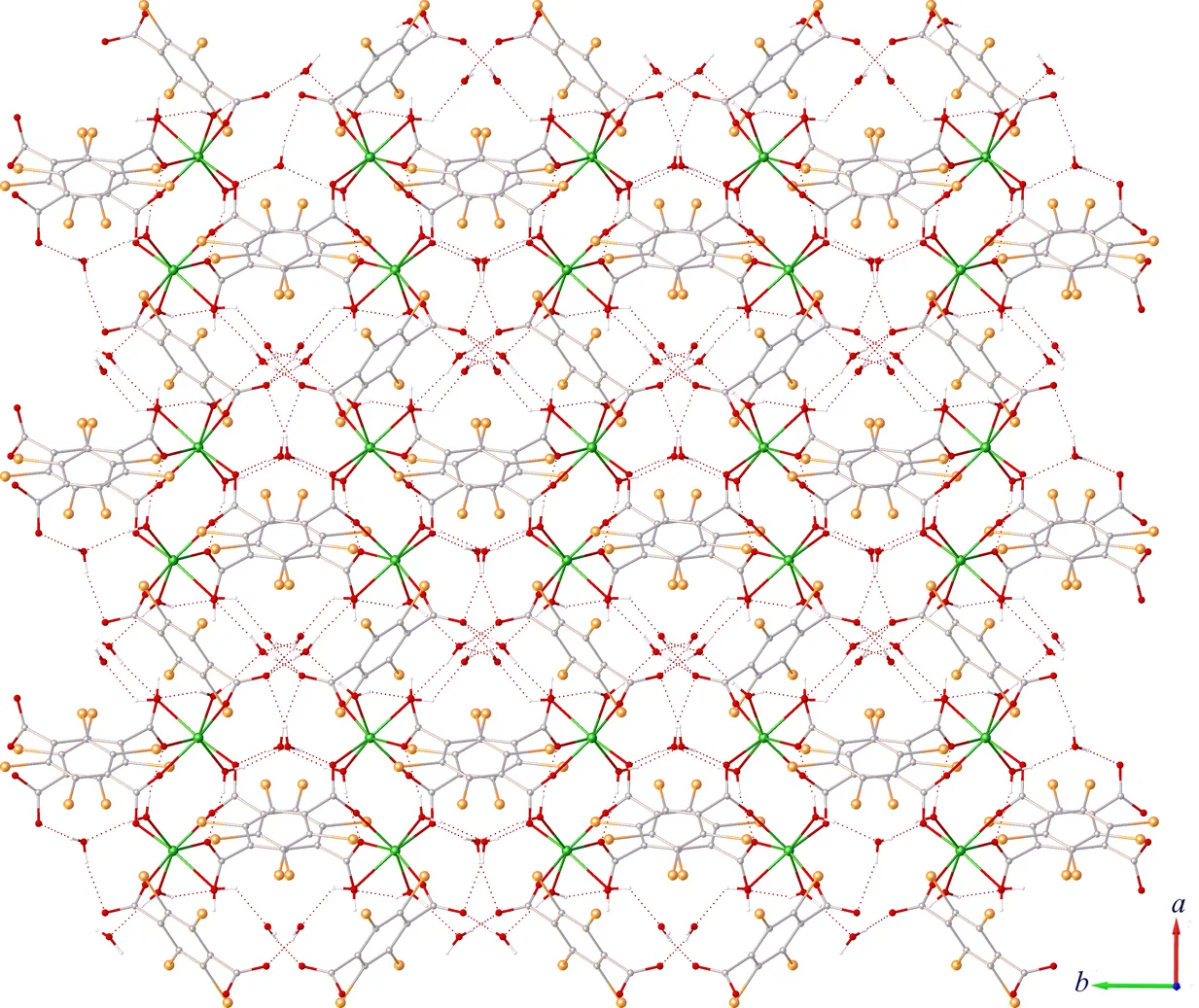
View of the three-dimensional framework of **1** along the *c* axis.

The asymmetric unit of **1** contains one Er^III^ atom, one and a half Br_4_bdc^2–^ ligands, three coordinated water mol­ecules, and three uncoordinated water mol­ecules. The Er^III^ ion adopts an [ErO_7_] coordination environment with four oxygen atoms from four different Br_4_bdc^2–^ ligands and three oxygen atoms from coordinated water mol­ecules (Fig. 1 ▸), displaying a distorted capped trigonal-prismatic geometry (CTPR-7; value 4.082) (*SHAPE 2.1*; Llunell *et al.*, 2013 ▸). The Er—O bond lengths fall in the range of 2.238 (2) to 2.347 (3) Å, mean 2.304 (2) Å. The O—Er—O bond angles range from 73.11 (8) to 155.44 (11)°. The fully deprotonated Br_4_bdc^2–^ ligand exhibits a *μ*_3_-κ*O*:κ*O′*:κ*O′′* bridging mode, linking three symmetry-related Er^III^ ions to propagate a two-dimensional sheet parallel to the *bc* plane (Fig. 2 ▸). Within this layer, the shortest Er⋯Er separation is 4.6625 (19) Å. The sheets are further inter­connected into a three-dimensional framework along the *c*-axis direction by the remaining Br_4_bdc^2–^ ligand, which resides on a crystallographic inversion centre and acts as a *μ*_2_-κ*O*:κ*O′* bridge (Fig. 3 ▸).

## 3. Supra­molecular features

**Figure 4:**
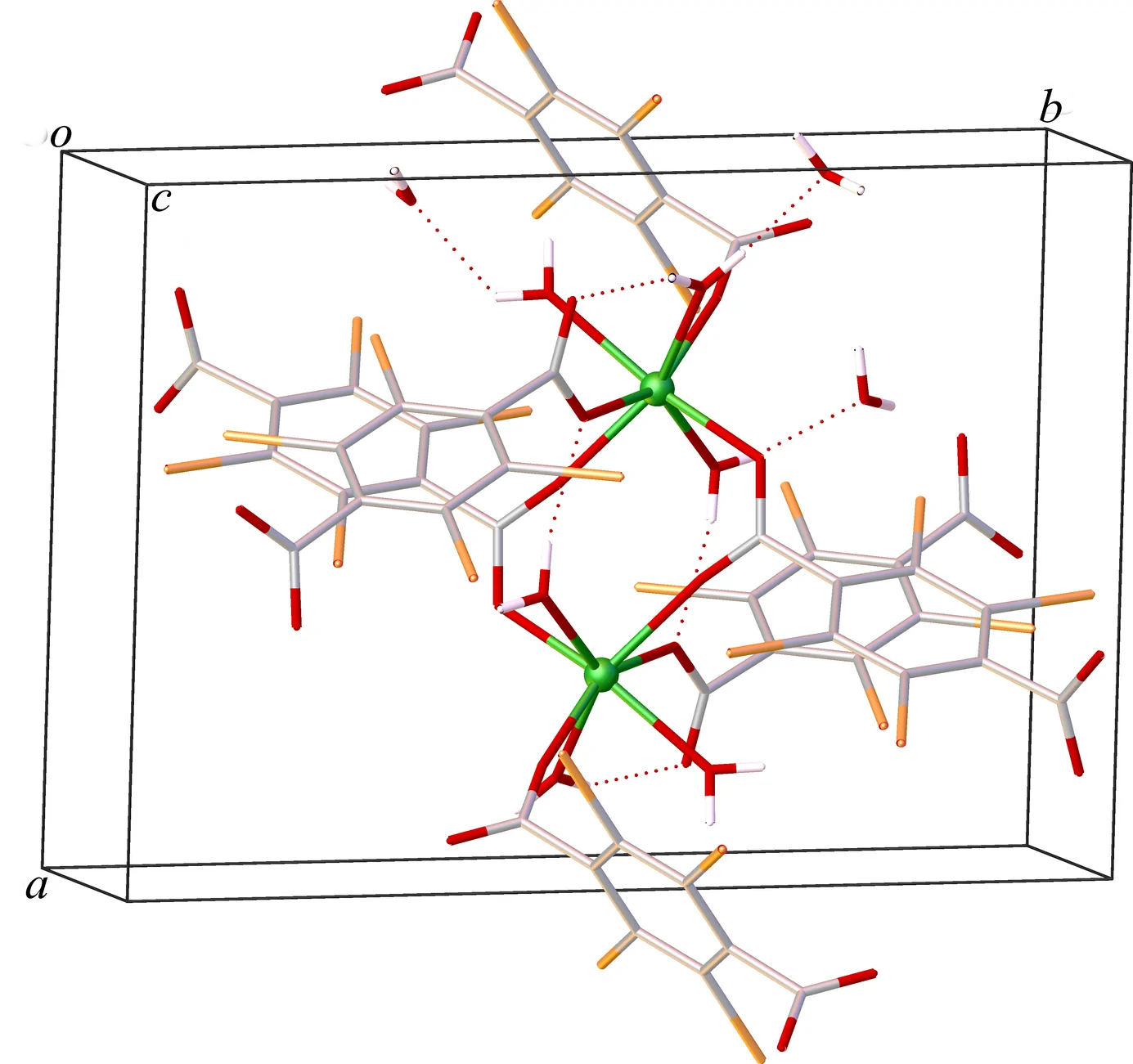
View of the O—H⋯O hydrogen bonds involving the carboxyl­ate groups of the Br_4_bdc^2–^ ligands and water mol­ecules of **1**. Hydrogen-bonding inter­actions are shown as dashed lines.

In the crystal structure, the coordinated water mol­ecules (O7*W*, O8*W* and O9*W*) act as hydrogen-bond donors, forming robust inter­molecular O—H⋯O hydrogen-bonding inter­actions with the water mol­ecules of crystallization (O10*W*, O11*W* and O12*W*) serving as the hydrogen-bond acceptors (Table 1 ▸). Moreover, the carboxyl­ate oxygen atoms of the Br_4_bdc^2–^ ligands serve as hydrogen-bond acceptors for the solvent water mol­ecules, contributing to the cohesion of the supra­molecular network (Fig. 4 ▸). The crystal packing is further consolidated by inter­molecular Br⋯Br and Br⋯O halogen bonding inter­actions (Table 2 ▸) occurring between adjacent Br_4_bdc^2–^ ligands. No significant π–π stacking inter­actions are observed between neighboring benzene rings, as the centroid-to-centroid separations fall well outside the typical 3.3–3.8 Å range characteristic of such supra­molecular contacts (Janiak, 2000 ▸).

## 4. Hirshfeld surface analysis

**Figure 5:**
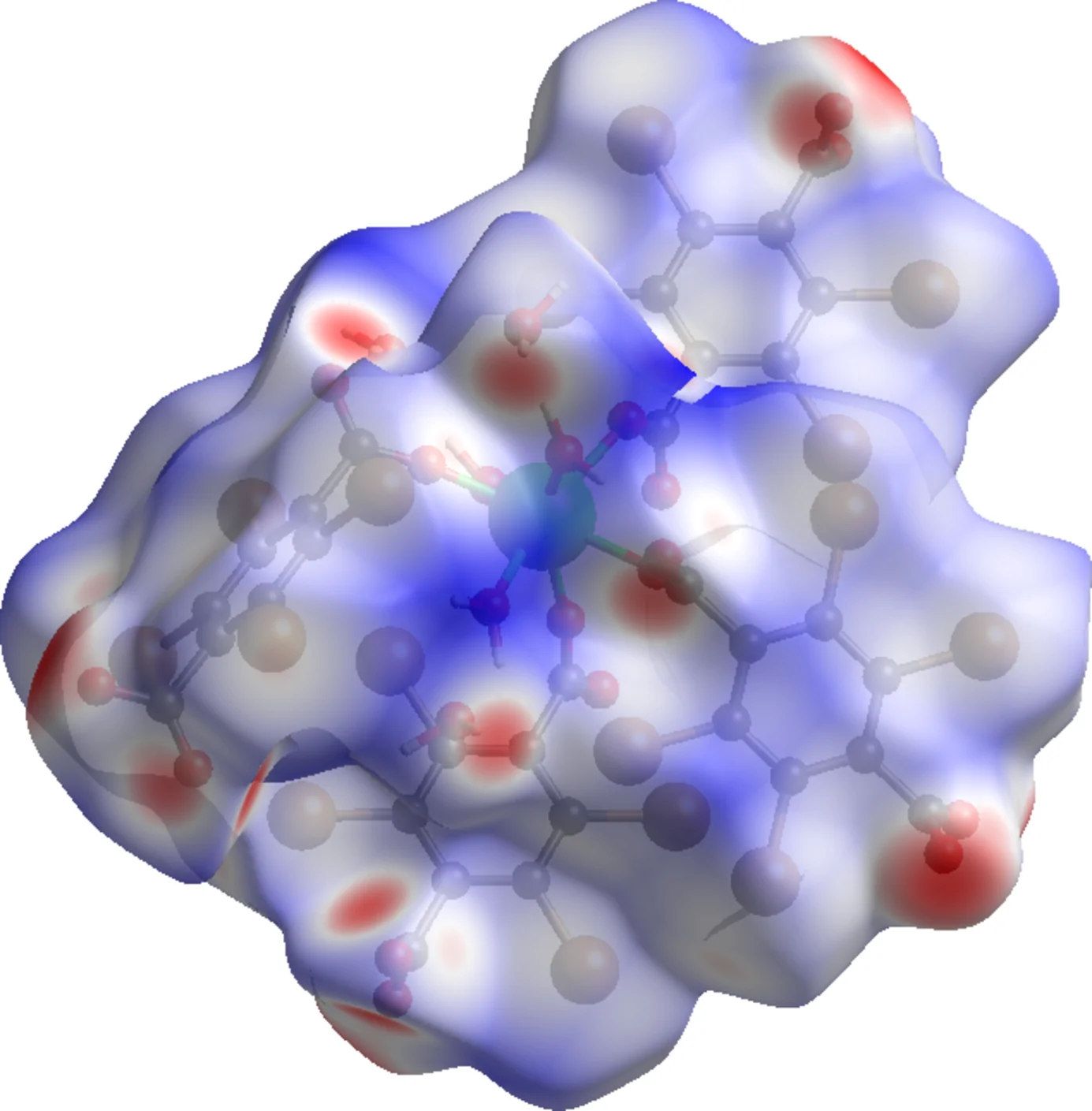
View of the Hirshfeld surface for **1** plotted over *d*_norm_.

**Figure 6:**
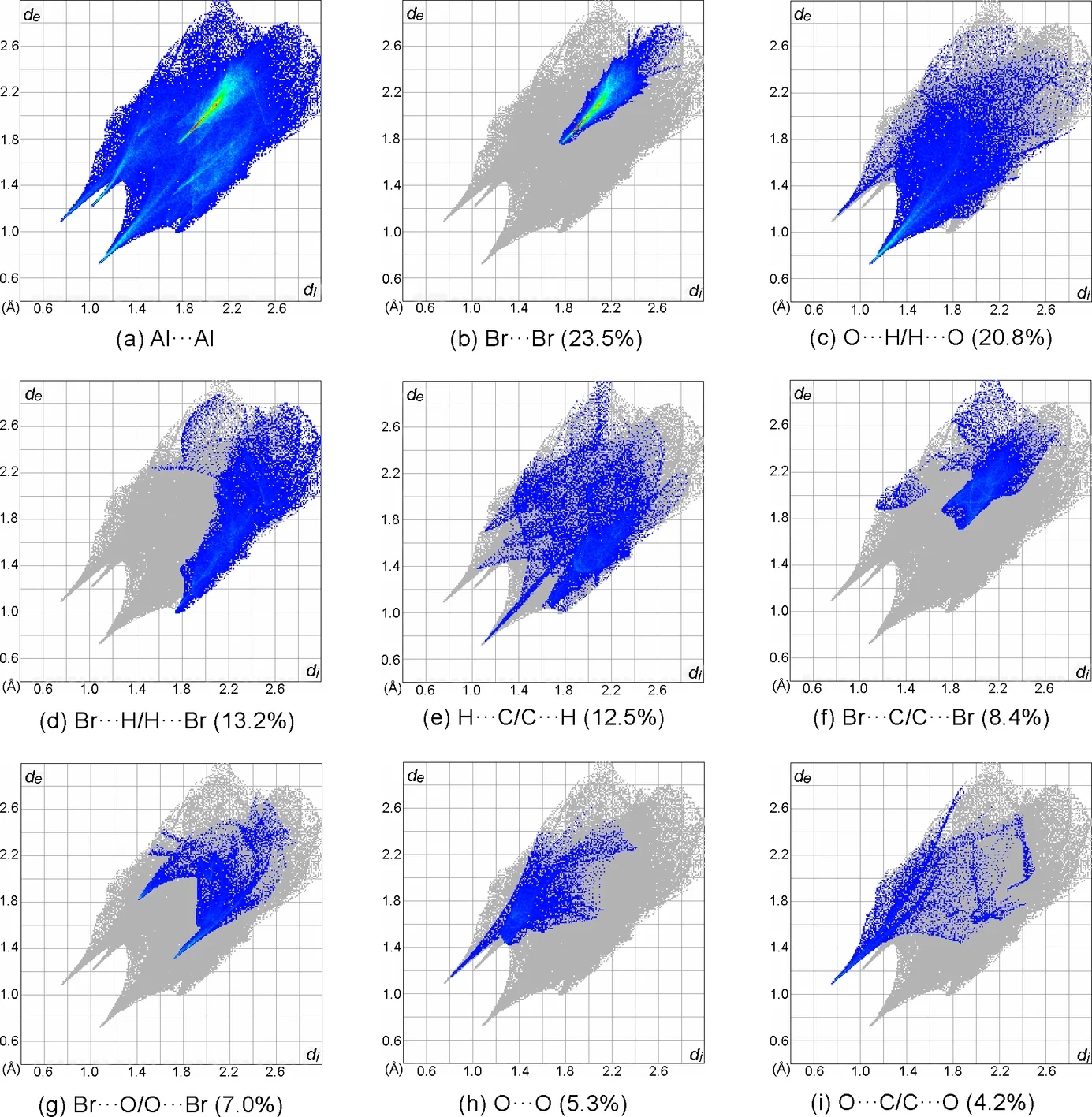
The two-dimensional fingerprint plots for **1**, showing (*a*) all inter­actions, and those delineated into (*b*) Br⋯Br, (*c*) O⋯H/H⋯O, (*d*) Br⋯H/H⋯Br, (*e*) H⋯C/C⋯H, (*f*) Br⋯C/C⋯Br, (*g*) Br⋯O/O⋯Br, (*h*) O⋯O, and (i) O⋯C/C⋯O inter­actions. The *d*_e_ and *d*_i_ values represent the closest external and inter­nal distances (Å), respectively, from a given point on the Hirshfeld surface.

A Hirshfeld surface analysis was carried out using *CrystalExplorer* (Spackman *et al.*, 2021 ▸) to examine the inter­molecular inter­actions in **1**. The 3D Hirshfeld surface mapped over *d*_norm_ highlights close inter­molecular contacts as red spots, whereas white and blue regions correspond to contacts close to and longer than the sum of the van der Waals radii, respectively (Fig. 5 ▸). The analysis of the two-dimensional fingerprint plots (Fig. 6 ▸) reveals that the largest contribution arises from Br⋯Br contacts (23.5%), indicating that close halogen⋯halogen contacts constitute a significant feature of the crystal packing. The O⋯H/H⋯O contacts (20.8%) correspond to the extensive network of O–H⋯O hydrogen bonds involving carboxyl­ate oxygen atoms, coordinated aqua ligands, and solvent water mol­ecules. Secondary contributions to the Hirshfeld surface area include Br⋯H/H⋯Br (13.2%) and H⋯C/C⋯H (12.5%) contacts, which further consolidate the supra­molecular architecture. Minor packing contributions are attributed to Br⋯C/C⋯Br (8.4%), Br⋯O/O⋯Br (7.0%), O⋯O (5.3%) and O⋯C/C⋯O (4.2%) contacts. Notably, C⋯C contacts account for only 0.7% of the Hirshfeld surface area, corroborating the absence of significant π–π stacking inter­actions within the crystal structure. Therefore, it can be concluded that halogen and hydrogen bonds are the major inter­molecular contacts that contribute to the consolidation of the crystal packing of **1**.

## 5. Database survey

A search of the Cambridge Structural Database (CSD version 6.01, September 2026 update; Groom *et al.*, 2016 ▸) for the structures of lanthanide complexes incorporating Br_4_bdc^2–^ ligands yielded 47 hits. Among these, thirteen structures, including SITROE and SITROE01 (Wei *et al.*, 2014 ▸; Smith *et al.*, 2019 ▸), BIQMAS, BIQMEW, BIQMIA, BIQMUM, BIQNAT, BIQNEX, BIQNIB, and BIQNOH (Smith *et al.*, 2019 ▸), FECFOM, FECFUS, and FECGAZ (Jiajaroen *et al.*, 2022 ▸), display concurrent *μ*_2_-κ*O*:κ*O′* and *μ*_3_-κ*O*:κ*O′*:κ*O′′* coordination modes of the Br_4_bdc^2–^ ligands to bind the *Ln*^III^ centres. Furthermore, the complexes BIQNUN, BIQPAV, BIQPEZ, BIQPID, and BIQPOJ (Smith *et al.*, 2019 ▸) exclusively exhibit the *μ*_3_-κ*O*:κ*O′*:κ*O′′* bridging mode, whereas NICZOQ (Lv *et al.*, 2013*a* ▸), WIPXUQ, and WIPYAX (Lv *et al.*, 2013*b* ▸) display only the *μ*_2_-κ*O*:κ*O′* coordination mode to bind the *Ln*^III^ cations.

## 6. Synthesis and crystallization

All starting materials and reagents were of analytical grade and were used as received without further purification. A mixture of Er(NO_3_)_3_·5H_2_O (44.3 mg, 0.1 mmol) and H_2_Br_4_bdc (48.2 mg, 0.1 mmol) was dissolved in a solvent mixture of methanol (1 mL) and distilled water (3 mL) in a 15 mL Teflon-lined reactor. The mixture solution was stirred at room temperature for 10 min, sealed in stainless steel autoclave, and heated under autogenous pressure in a temperature-controlled oven at 373 K for 48 h. After the autoclave had cooled slowly to ambient temperature, the reaction mixture was filtered, and the filtrate was transferred to a glass vial. Slow evaporation of the mother liquor at room temperature over approximately one week yielded pink block-shaped crystals. Yield: 12.4 mg, 28% based on the Er(NO_3_)_3_·5H_2_O.

## 7. Refinement

Crystal data, data collection and structure refinement details are summarized in Table 3 ▸. The hydrogen atoms were located from difference electron-density maps. H atoms bonded to O atoms were refined with distance restraints O—H = 0.84 ± 0.02 Å and with *U*_iso_(H) = 1.5*U*_eq_(O) for H9*A*/*B* and H10*A*/*B* and independently refined for eight other atoms.

## Supplementary Material

Crystal structure: contains datablock(s) I. DOI: 10.1107/S2056989026010698/vm2337sup1.cif

Structure factors: contains datablock(s) I. DOI: 10.1107/S2056989026010698/vm2337Isup2.hkl

CCDC reference: 2595517

Additional supporting information:  crystallographic information; 3D view; checkCIF report

## Figures and Tables

**Table 1:** Hydrogen-bond geometry (Å, °)

*D*—H⋯*A*	*D*—H	H⋯*A*	*D*⋯*A*	*D*—H⋯*A*
O7—H7*A*⋯O11	0.86 (3)	2.06 (3)	2.892 (4)	165 (5)
O7—H7*B*⋯O4^i^	0.84 (3)	2.10 (3)	2.931 (4)	168 (4)
O8—H8*A*⋯O12	0.84 (3)	1.86 (3)	2.705 (4)	174 (3)
O8—H8*B*⋯O3^ii^	0.82 (4)	1.96 (3)	2.703 (3)	152 (5)
O9—H9*A*⋯O10	0.84 (2)	2.05 (4)	2.643 (5)	128 (4)
O10—H10*A*⋯O12^iii^	0.85 (3)	2.00 (5)	2.808 (4)	160 (5)
O10—H10*B*⋯O6^iii^	0.84 (5)	2.12 (6)	2.948 (5)	171 (6)
O11—H11*A*⋯O6	0.84 (3)	2.18 (4)	2.989 (4)	161 (5)
O11—H11*B*⋯O2^i^	0.84 (5)	2.31 (5)	3.139 (4)	174 (6)
O12—H12*A*⋯O3^iv^	0.84 (4)	2.06 (4)	2.879 (4)	164 (4)
O12—H12*B*⋯O6^v^	0.84 (4)	1.99 (4)	2.832 (3)	177 (4)

Symmetry codes: (i) 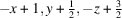; (ii) 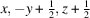; (iii) 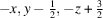; (iv) 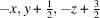; (v) 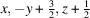.

**Table 2:** Br⋯*X* halogen-bond geometry (Å, °)

C—Br⋯*X*	Br⋯*X*	C—Br⋯*X*	Type
C4—Br2⋯Br2^i^	3.7489 (14)	129.40 (7)	I
C6—Br3⋯Br6	3.8861 (11)	134.30 (8)	II
C7—Br4⋯Br2^ii^	3.7048 (13)	120.25 (8)	I
C7—Br4⋯Br5	3.5390 (15)	161.06 (8)	II
C11—Br5⋯Br1^ii^	3.5739 (15)	174.31 (9)	II
C3—Br1⋯O11^iii^	3.054 (3)	160.04 (10)	
C12—Br6⋯O3^iv^	3.265 (3)	175.75 (9)	

Symmetry codes: (i) 1 − *x*, −*y*, 2 − *z*; (ii) 1 − *x*, 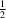 + *y*, 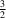 − *z*; (iii) 1 − *x*, 1 − *y*, 2 − *z*; (iv) *x*, 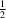 − *y*, −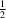 + *z*.

**Table 3:** Experimental details

Crystal data	
Chemical formula	[Er(C_8_Br_4_O_4_)_1.5_(H_2_O)_3_]·3H_2_O
*M*_r_	994.94
Crystal system, space group	Monoclinic, *P*2_1_/*c*
Temperature (K)	296
*a*, *b*, *c* (Å)	12.711 (5), 15.839 (6), 12.906 (5)
β (°)	113.171 (15)
*V* (Å^3^)	2388.9 (17)
*Z*	4
Radiation type	Mo *K*α
μ (mm^−1^)	13.60
Crystal size (mm)	0.14 × 0.12 × 0.1

Data collection	
Diffractometer	Bruker D8 QUEST CMOS PHOTON II
Absorption correction	Multi-scan (*SADABS*; Krause *et al.*, 2015 ▸)
*T*_min_, *T*_max_	0.571, 0.746
No. of measured, independent and observed [*I* > 2σ(*I*)] reflections	98614, 5854, 5331
*R*_int_	0.055
(sin θ/λ)_max_ (Å^−1^)	0.664

Refinement	
*R*[*F*^2^ > 2σ(*F*^2^)], *wR*(*F*^2^), *S*	0.019, 0.046, 1.17
No. of reflections	5854
No. of parameters	324
No. of restraints	17
H-atom treatment	Only H-atom coordinates refined
Δρ_max_, Δρ_min_ (e Å^−3^)	0.50, −1.21

Computer programs: *APEX6* and *SAINT* (Bruker, 2023 ▸), *SHELXT2014/5* (Sheldrick, 2015*a* ▸), *SHELXL2018/3* (Sheldrick, 2015*b* ▸) and *OLEX2* (Dolomanov *et al.*, 2009 ▸).

## supplementary crystallographic information

### Poly[[triaqua(µ3-2,3,5,6-tetrabromobenzene-1,4-dicarboxylato)hemi(µ2-2,3,5,6-tetrabromobenzene-1,4-dicarboxylato)erbium(III)] trihydrate] Crystal data

**Table:**

[Er(C_8_Br_4_O_4_)_1.5_(H_2_O)_3_]·3H_2_O	*F*(000) = 1832
*M**_r_* = 994.94	*D*_x_ = 2.766 Mg m^−^^3^
Monoclinic, *P*2_1_/*c*	Mo *K*α radiation, λ = 0.71073 Å
*a* = 12.711 (5) Å	Cell parameters from 8607 reflections
*b* = 15.839 (6) Å	θ = 3.1–28.1°
*c* = 12.906 (5) Å	µ = 13.60 mm^−^^1^
β = 113.171 (15)°	*T* = 296 K
*V* = 2388.9 (17) Å^3^	Block, pink
*Z* = 4	0.14 × 0.12 × 0.1 mm

### Poly[[triaqua(µ3-2,3,5,6-tetrabromobenzene-1,4-dicarboxylato)hemi(µ2-2,3,5,6-tetrabromobenzene-1,4-dicarboxylato)erbium(III)] trihydrate] Data collection

**Table:**

Bruker D8 QUEST CMOS PHOTON II diffractometer	5854 independent reflections
Radiation source: sealed x-ray tube	5331 reflections with *I* > 2σ(*I*)
Graphite monochromator	*R*_int_ = 0.055
Detector resolution: 7.39 pixels mm^-1^	θ_max_ = 28.2°, θ_min_ = 3.1°
φ and ω scans	*h* = −16→16
Absorption correction: multi-scan (SADABS; Krause *et al.*, 2015)	*k* = −21→21
*T*_min_ = 0.571, *T*_max_ = 0.746	*l* = −17→17
98614 measured reflections	

### Poly[[triaqua(µ3-2,3,5,6-tetrabromobenzene-1,4-dicarboxylato)hemi(µ2-2,3,5,6-tetrabromobenzene-1,4-dicarboxylato)erbium(III)] trihydrate] Refinement

**Table:**

Refinement on *F*^2^	17 restraints
Least-squares matrix: full	Hydrogen site location: difference Fourier map
*R*[*F*^2^ > 2σ(*F*^2^)] = 0.019	Only H-atom coordinates refined
*wR*(*F*^2^) = 0.046	*w* = 1/[σ^2^(*F*_o_^2^) + (0.0183*P*)^2^ + 2.0786*P*] where *P* = (*F*_o_^2^ + 2*F*_c_^2^)/3
*S* = 1.17	(Δ/σ)_max_ = 0.002
5854 reflections	Δρ_max_ = 0.50 e Å^−^^3^
324 parameters	Δρ_min_ = −1.21 e Å^−^^3^

### Poly[[triaqua(µ3-2,3,5,6-tetrabromobenzene-1,4-dicarboxylato)hemi(µ2-2,3,5,6-tetrabromobenzene-1,4-dicarboxylato)erbium(III)] trihydrate] Special details

**Table:**

Geometry. All esds (except the esd in the dihedral angle between two l.s. planes) are estimated using the full covariance matrix. The cell esds are taken into account individually in the estimation of esds in distances, angles and torsion angles; correlations between esds in cell parameters are only used when they are defined by crystal symmetry. An approximate (isotropic) treatment of cell esds is used for estimating esds involving l.s. planes.

### Poly[[triaqua(µ3-2,3,5,6-tetrabromobenzene-1,4-dicarboxylato)hemi(µ2-2,3,5,6-tetrabromobenzene-1,4-dicarboxylato)erbium(III)] trihydrate] Fractional atomic coordinates and isotropic or equivalent isotropic displacement parameters (Å^2^)

**Table:**

	*x*	*y*	*z*	*U*_iso_*/*U*_eq_	
Er1	0.30558 (2)	0.53186 (2)	0.91422 (2)	0.01547 (4)	
Br1	0.53394 (2)	0.20275 (2)	1.02462 (2)	0.02423 (7)	
Br2	0.40531 (3)	0.04112 (2)	0.85749 (3)	0.02935 (7)	
Br3	0.22167 (3)	0.26274 (2)	0.48667 (2)	0.02803 (7)	
Br4	0.34474 (3)	0.42516 (2)	0.65020 (3)	0.03093 (7)	
Br5	0.21775 (3)	0.60657 (2)	0.48962 (3)	0.03010 (7)	
Br6	0.08858 (3)	0.45787 (2)	0.29799 (3)	0.03033 (7)	
O1	0.4355 (2)	0.43322 (13)	0.91634 (18)	0.0320 (5)	
O2	0.60068 (18)	0.37370 (14)	0.94057 (17)	0.0273 (5)	
O3	0.16920 (18)	0.07189 (14)	0.5898 (2)	0.0306 (5)	
O4	0.33523 (17)	0.05543 (13)	0.56882 (17)	0.0234 (4)	
O5	0.18810 (17)	0.60262 (14)	0.76286 (17)	0.0274 (5)	
O6	0.09196 (19)	0.70465 (13)	0.64610 (18)	0.0297 (5)	
O7	0.42133 (19)	0.60002 (14)	0.83626 (18)	0.0256 (4)	
H7A	0.406 (4)	0.6462 (18)	0.799 (3)	0.052 (13)*	
H7B	0.4929 (17)	0.594 (3)	0.869 (3)	0.045 (12)*	
O8	0.1619 (2)	0.56996 (15)	0.9707 (2)	0.0317 (5)	
H8A	0.116 (3)	0.6106 (17)	0.961 (3)	0.031 (10)*	
H8B	0.146 (4)	0.536 (2)	1.010 (3)	0.056 (14)*	
O9	0.1742 (3)	0.42709 (17)	0.8218 (3)	0.0565 (9)	
H9A	0.177 (3)	0.3743 (12)	0.821 (4)	0.085*	
H9B	0.1023 (18)	0.429 (2)	0.783 (4)	0.085*	
O10	0.0444 (3)	0.2919 (2)	0.7472 (3)	0.0723 (11)	
H10A	0.021 (5)	0.274 (4)	0.680 (2)	0.108*	
H10B	0.000 (4)	0.271 (4)	0.774 (4)	0.108*	
O11	0.3362 (2)	0.7592 (2)	0.7251 (2)	0.0456 (7)	
H11A	0.2668 (19)	0.753 (3)	0.713 (4)	0.057 (14)*	
H11B	0.348 (5)	0.791 (3)	0.679 (4)	0.10 (2)*	
O12	0.0093 (2)	0.69470 (16)	0.9489 (2)	0.0341 (5)	
H12A	−0.045 (3)	0.664 (2)	0.948 (4)	0.045 (12)*	
H12B	0.032 (4)	0.726 (3)	1.006 (3)	0.076 (17)*	
C1	0.4937 (2)	0.37515 (17)	0.8996 (2)	0.0194 (5)	
C2	0.4312 (2)	0.30194 (16)	0.8252 (2)	0.0159 (5)	
C3	0.4444 (2)	0.21992 (16)	0.8697 (2)	0.0159 (5)	
C4	0.3897 (2)	0.15226 (16)	0.8005 (2)	0.0174 (5)	
C5	0.3237 (2)	0.16509 (16)	0.6859 (2)	0.0167 (5)	
C6	0.3104 (2)	0.24731 (17)	0.6421 (2)	0.0183 (5)	
C7	0.3628 (2)	0.31511 (16)	0.7112 (2)	0.0174 (5)	
C8	0.2701 (2)	0.09094 (17)	0.6086 (2)	0.0179 (5)	
C9	0.1154 (2)	0.62976 (18)	0.6702 (2)	0.0196 (5)	
C10	0.0550 (2)	0.56271 (18)	0.5812 (2)	0.0194 (5)	
C11	0.0911 (2)	0.54480 (18)	0.4947 (2)	0.0212 (6)	
C12	0.0374 (2)	0.48307 (18)	0.4142 (2)	0.0206 (5)	

### Poly[[triaqua(µ3-2,3,5,6-tetrabromobenzene-1,4-dicarboxylato)hemi(µ2-2,3,5,6-tetrabromobenzene-1,4-dicarboxylato)erbium(III)] trihydrate] Atomic displacement parameters (Å^2^)

**Table:**

	*U*^11^	*U*^22^	*U*^33^	*U*^12^	*U*^13^	*U*^23^
Er1	0.01698 (6)	0.01306 (6)	0.01333 (6)	−0.00005 (4)	0.00272 (4)	0.00077 (4)
Br1	0.02666 (15)	0.02368 (14)	0.01605 (13)	0.00183 (11)	0.00162 (11)	0.00077 (10)
Br2	0.03810 (17)	0.01532 (13)	0.02970 (16)	0.00103 (12)	0.00805 (13)	0.00287 (11)
Br3	0.02991 (16)	0.03137 (16)	0.01536 (13)	−0.00545 (12)	0.00091 (11)	0.00117 (11)
Br4	0.04617 (19)	0.01730 (14)	0.02426 (15)	−0.00146 (13)	0.00843 (13)	0.00352 (11)
Br5	0.02511 (15)	0.03419 (17)	0.03360 (16)	−0.01130 (12)	0.01434 (13)	−0.00158 (13)
Br6	0.02646 (15)	0.04207 (18)	0.02597 (15)	−0.00518 (13)	0.01410 (12)	−0.00478 (13)
O1	0.0503 (15)	0.0217 (11)	0.0255 (11)	0.0102 (10)	0.0164 (10)	−0.0037 (9)
O2	0.0253 (11)	0.0292 (11)	0.0241 (10)	−0.0101 (9)	0.0062 (9)	−0.0075 (9)
O3	0.0215 (11)	0.0304 (12)	0.0410 (13)	−0.0077 (9)	0.0134 (10)	−0.0145 (10)
O4	0.0229 (10)	0.0239 (10)	0.0236 (10)	−0.0031 (8)	0.0093 (8)	−0.0105 (8)
O5	0.0223 (11)	0.0307 (11)	0.0192 (10)	−0.0027 (9)	−0.0025 (8)	0.0075 (8)
O6	0.0333 (12)	0.0235 (11)	0.0253 (11)	0.0019 (9)	0.0039 (9)	0.0034 (9)
O7	0.0238 (11)	0.0264 (11)	0.0267 (11)	0.0011 (9)	0.0101 (9)	0.0051 (9)
O8	0.0308 (12)	0.0286 (12)	0.0410 (13)	0.0127 (10)	0.0198 (11)	0.0143 (10)
O9	0.0589 (19)	0.0343 (15)	0.0499 (17)	−0.0264 (14)	−0.0068 (14)	0.0031 (13)
O10	0.094 (3)	0.075 (2)	0.0420 (17)	−0.057 (2)	0.0201 (17)	−0.0184 (16)
O11	0.0333 (15)	0.0563 (18)	0.0401 (15)	−0.0100 (13)	0.0067 (12)	0.0124 (13)
O12	0.0307 (13)	0.0340 (13)	0.0385 (14)	0.0013 (10)	0.0145 (11)	−0.0060 (11)
C1	0.0285 (15)	0.0143 (12)	0.0145 (12)	−0.0036 (11)	0.0075 (11)	−0.0018 (10)
C2	0.0153 (12)	0.0151 (12)	0.0170 (12)	0.0006 (9)	0.0058 (10)	−0.0041 (10)
C3	0.0151 (12)	0.0173 (12)	0.0141 (12)	0.0040 (10)	0.0044 (10)	−0.0013 (9)
C4	0.0189 (13)	0.0125 (12)	0.0206 (13)	0.0012 (10)	0.0076 (10)	−0.0001 (10)
C5	0.0174 (12)	0.0158 (12)	0.0168 (12)	−0.0017 (10)	0.0067 (10)	−0.0054 (10)
C6	0.0192 (13)	0.0202 (13)	0.0135 (12)	0.0000 (10)	0.0044 (10)	−0.0007 (10)
C7	0.0209 (13)	0.0142 (12)	0.0175 (12)	0.0010 (10)	0.0079 (10)	0.0003 (9)
C8	0.0193 (13)	0.0170 (12)	0.0157 (12)	−0.0014 (10)	0.0051 (10)	−0.0035 (10)
C9	0.0145 (12)	0.0249 (14)	0.0181 (13)	−0.0010 (10)	0.0049 (10)	0.0052 (11)
C10	0.0150 (12)	0.0217 (13)	0.0169 (13)	0.0010 (10)	0.0012 (10)	0.0045 (10)
C11	0.0153 (13)	0.0249 (14)	0.0219 (14)	−0.0029 (11)	0.0055 (11)	0.0042 (11)
C12	0.0192 (13)	0.0258 (14)	0.0158 (12)	−0.0005 (11)	0.0056 (10)	0.0046 (10)

### Poly[[triaqua(µ3-2,3,5,6-tetrabromobenzene-1,4-dicarboxylato)hemi(µ2-2,3,5,6-tetrabromobenzene-1,4-dicarboxylato)erbium(III)] trihydrate] Geometric parameters (Å, º)

**Table:**

Er1—O1	2.266 (2)	O8—H8B	0.820 (19)
Er1—O2^i^	2.326 (2)	O9—H9A	0.837 (18)
Er1—O4^ii^	2.333 (2)	O9—H9B	0.852 (18)
Er1—O5	2.238 (2)	O10—H10A	0.845 (14)
Er1—O7	2.347 (2)	O10—H10B	0.842 (14)
Er1—O8	2.297 (2)	O11—H11A	0.838 (19)
Er1—O9	2.323 (3)	O11—H11B	0.84 (2)
Br1—C3	1.891 (3)	O12—H12A	0.848 (19)
Br2—C4	1.888 (3)	O12—H12B	0.835 (19)
Br3—C6	1.891 (3)	C1—C2	1.516 (4)
Br4—C7	1.889 (3)	C2—C3	1.404 (4)
Br5—C11	1.906 (3)	C2—C7	1.399 (4)
Br6—C12	1.898 (3)	C3—C4	1.394 (4)
O1—C1	1.251 (3)	C4—C5	1.398 (4)
O2—C1	1.250 (4)	C5—C6	1.403 (4)
O3—C8	1.245 (3)	C5—C8	1.518 (4)
O4—C8	1.265 (3)	C6—C7	1.387 (4)
O5—C9	1.265 (3)	C9—C10	1.531 (4)
O6—C9	1.233 (3)	C10—C11	1.394 (4)
O7—H7A	0.853 (19)	C10—C12^iii^	1.401 (4)
O7—H7B	0.843 (19)	C11—C12	1.394 (4)
O8—H8A	0.841 (18)		
			
O1—Er1—O2^i^	107.08 (8)	O1—C1—C2	118.3 (3)
O1—Er1—O4^ii^	73.11 (8)	O2—C1—O1	124.0 (3)
O1—Er1—O7	75.24 (9)	O2—C1—C2	117.7 (2)
O1—Er1—O8	147.57 (8)	C3—C2—C1	120.0 (2)
O1—Er1—O9	83.53 (11)	C7—C2—C1	120.4 (2)
O2^i^—Er1—O4^ii^	80.38 (8)	C7—C2—C3	119.6 (2)
O2^i^—Er1—O7	80.84 (8)	C2—C3—Br1	119.45 (19)
O4^ii^—Er1—O7	136.20 (8)	C4—C3—Br1	120.6 (2)
O5—Er1—O1	126.07 (8)	C4—C3—C2	120.0 (2)
O5—Er1—O2^i^	109.61 (8)	C3—C4—Br2	121.2 (2)
O5—Er1—O4^ii^	150.60 (8)	C3—C4—C5	120.5 (2)
O5—Er1—O7	73.20 (8)	C5—C4—Br2	118.37 (19)
O5—Er1—O8	78.73 (9)	C4—C5—C6	119.2 (2)
O5—Er1—O9	79.14 (9)	C4—C5—C8	120.7 (2)
O8—Er1—O2^i^	78.15 (9)	C6—C5—C8	120.1 (2)
O8—Er1—O4^ii^	76.44 (8)	C5—C6—Br3	118.34 (19)
O8—Er1—O7	136.54 (8)	C7—C6—Br3	121.1 (2)
O8—Er1—O9	81.35 (12)	C7—C6—C5	120.5 (2)
O9—Er1—O2^i^	155.44 (11)	C2—C7—Br4	119.9 (2)
O9—Er1—O4^ii^	81.79 (9)	C6—C7—Br4	119.9 (2)
O9—Er1—O7	123.64 (11)	C6—C7—C2	120.2 (2)
C1—O1—Er1	169.2 (2)	O3—C8—O4	127.0 (2)
C1—O2—Er1^i^	117.13 (18)	O3—C8—C5	118.5 (2)
C8—O4—Er1^iv^	134.39 (18)	O4—C8—C5	114.5 (2)
C9—O5—Er1	169.8 (2)	O5—C9—C10	116.0 (2)
Er1—O7—H7A	127 (3)	O6—C9—O5	125.4 (3)
Er1—O7—H7B	118 (3)	O6—C9—C10	118.6 (2)
H7A—O7—H7B	109 (4)	C11—C10—C9	121.0 (2)
Er1—O8—H8A	137 (2)	C11—C10—C12^iii^	118.3 (3)
Er1—O8—H8B	116 (3)	C12^iii^—C10—C9	120.6 (2)
H8A—O8—H8B	107 (4)	C10—C11—Br5	118.2 (2)
Er1—O9—H9A	134 (2)	C12—C11—Br5	120.5 (2)
Er1—O9—H9B	132 (2)	C12—C11—C10	121.3 (3)
H9A—O9—H9B	94 (3)	C10^iii^—C12—Br6	118.6 (2)
H10A—O10—H10B	106 (4)	C11—C12—Br6	121.1 (2)
H11A—O11—H11B	114 (5)	C11—C12—C10^iii^	120.3 (3)
H12A—O12—H12B	111 (4)		
			
Er1—O1—C1—O2	150.8 (10)	C1—C2—C7—Br4	−1.6 (4)
Er1—O1—C1—C2	−29.2 (13)	C1—C2—C7—C6	176.8 (2)
Er1^i^—O2—C1—O1	13.9 (4)	C2—C3—C4—Br2	−179.92 (19)
Er1^i^—O2—C1—C2	−166.12 (17)	C2—C3—C4—C5	1.6 (4)
Er1^iv^—O4—C8—O3	19.4 (5)	C3—C2—C7—Br4	−179.82 (19)
Er1^iv^—O4—C8—C5	−159.79 (18)	C3—C2—C7—C6	−1.4 (4)
Er1—O5—C9—O6	174.9 (9)	C3—C4—C5—C6	−1.9 (4)
Er1—O5—C9—C10	−6.6 (13)	C3—C4—C5—C8	176.1 (2)
Br1—C3—C4—Br2	−1.4 (3)	C4—C5—C6—Br3	179.5 (2)
Br1—C3—C4—C5	−179.9 (2)	C4—C5—C6—C7	0.6 (4)
Br2—C4—C5—C6	179.6 (2)	C4—C5—C8—O3	95.4 (3)
Br2—C4—C5—C8	−2.5 (3)	C4—C5—C8—O4	−85.4 (3)
Br3—C6—C7—Br4	0.6 (3)	C5—C6—C7—Br4	179.5 (2)
Br3—C6—C7—C2	−177.8 (2)	C5—C6—C7—C2	1.1 (4)
Br5—C11—C12—Br6	0.4 (3)	C6—C5—C8—O3	−86.7 (3)
Br5—C11—C12—C10^iii^	179.4 (2)	C6—C5—C8—O4	92.6 (3)
O1—C1—C2—C3	−117.9 (3)	C7—C2—C3—Br1	−178.5 (2)
O1—C1—C2—C7	64.0 (4)	C7—C2—C3—C4	0.1 (4)
O2—C1—C2—C3	62.2 (3)	C8—C5—C6—Br3	1.5 (3)
O2—C1—C2—C7	−116.0 (3)	C8—C5—C6—C7	−177.4 (2)
O5—C9—C10—C11	−98.5 (3)	C9—C10—C11—Br5	−0.3 (4)
O5—C9—C10—C12^iii^	80.6 (3)	C9—C10—C11—C12	179.1 (3)
O6—C9—C10—C11	80.1 (4)	C10—C11—C12—Br6	−179.1 (2)
O6—C9—C10—C12^iii^	−100.8 (3)	C10—C11—C12—C10^iii^	0.0 (5)
C1—C2—C3—Br1	3.3 (3)	C12^iii^—C10—C11—Br5	−179.5 (2)
C1—C2—C3—C4	−178.1 (2)	C12^iii^—C10—C11—C12	0.0 (5)

Symmetry codes: (i) −*x*+1, −*y*+1, −*z*+2; (ii) *x*, −*y*+1/2, *z*+1/2; (iii) −*x*, −*y*+1, −*z*+1; (iv) *x*, −*y*+1/2, *z*−1/2.

### Poly[[triaqua(µ3-2,3,5,6-tetrabromobenzene-1,4-dicarboxylato)hemi(µ2-2,3,5,6-tetrabromobenzene-1,4-dicarboxylato)erbium(III)] trihydrate] Hydrogen-bond geometry (Å, º)

**Table:**

*D*—H···*A*	*D*—H	H···*A*	*D*···*A*	*D*—H···*A*
O7—H7*A*···O11	0.86 (3)	2.06 (3)	2.892 (4)	165 (5)
O7—H7*B*···O4^v^	0.84 (3)	2.10 (3)	2.931 (4)	168 (4)
O8—H8*A*···O12	0.84 (3)	1.86 (3)	2.705 (4)	174 (3)
O8—H8*B*···O3^ii^	0.82 (4)	1.96 (3)	2.703 (3)	152 (5)
O9—H9*A*···O10	0.84 (2)	2.05 (4)	2.643 (5)	128 (4)
O10—H10*A*···O12^vi^	0.85 (3)	2.00 (5)	2.808 (4)	160 (5)
O10—H10*B*···O6^vi^	0.84 (5)	2.12 (6)	2.948 (5)	171 (6)
O11—H11*A*···O6	0.84 (3)	2.18 (4)	2.989 (4)	161 (5)
O11—H11*B*···O2^v^	0.84 (5)	2.31 (5)	3.139 (4)	174 (6)
O12—H12*A*···O3^vii^	0.84 (4)	2.06 (4)	2.879 (4)	164 (4)
O12—H12*B*···O6^viii^	0.84 (4)	1.99 (4)	2.832 (3)	177 (4)

Symmetry codes: (ii) *x*, −*y*+1/2, *z*+1/2; (v) −*x*+1, *y*+1/2, −*z*+3/2; (vi) −*x*, *y*−1/2, −*z*+3/2; (vii) −*x*, *y*+1/2, −*z*+3/2; (viii) *x*, −*y*+3/2, *z*+1/2.
